# pGenN, a Gene Normalization Tool for Plant Genes and Proteins in Scientific Literature

**DOI:** 10.1371/journal.pone.0135305

**Published:** 2015-08-10

**Authors:** Ruoyao Ding, Cecilia N. Arighi, Jung-Youn Lee, Cathy H. Wu, K. Vijay-Shanker

**Affiliations:** 1 Department of Computer and Information Sciences, University of Delaware, Newark, Delaware, United States of America; 2 Center for Bioinformatics and Computational Biology, University of Delaware, Newark, Delaware, United States of America; 3 Department of Plant and Soil Sciences, University of Delaware, Newark, Delaware, United States of America; National Center for Biotechnology Information, UNITED STATES

## Abstract

**Background:**

Automatically detecting gene/protein names in the literature and connecting them to databases records, also known as gene normalization, provides a means to structure the information buried in free-text literature. Gene normalization is critical for improving the coverage of annotation in the databases, and is an essential component of many text mining systems and database curation pipelines.

**Methods:**

In this manuscript, we describe a gene normalization system specifically tailored for plant species, called pGenN (pivot-based Gene Normalization). The system consists of three steps: dictionary-based gene mention detection, species assignment, and intra species normalization. We have developed new heuristics to improve each of these phases.

**Results:**

We evaluated the performance of pGenN on an in-house expertly annotated corpus consisting of 104 plant relevant abstracts. Our system achieved an F-value of 88.9% (Precision 90.9% and Recall 87.2%) on this corpus, outperforming state-of-art systems presented in BioCreative III. We have processed over 440,000 plant-related Medline abstracts using pGenN. The gene normalization results are stored in a local database for direct query from the pGenN web interface (proteininformationresource.org/pgenn/). The annotated literature corpus is also publicly available through the PIR text mining portal (proteininformationresource.org/iprolink/).

## Introduction

A major focus of modern biological research is to link big data to knowledge [1], which requires tools to provide structure to information in unstructured sources such as the scientific literature. One barrier to structured representation of information in the literature is the highly complex nature of the nomenclature of genes and proteins. Multiple names and symbols are frequently used to refer to the same entity, and conversely, a given name or symbol can refer to multiple entities. In order to comprehensively annotate gene/protein records and to support queries from biologists from a variety of backgrounds who may use different names to refer to a gene/protein of interest, curators of knowledge bases, such as UniProt [2], need to capture the full range of names and symbols by which a protein/gene is known. Automatic detection of gene/protein names in the literature and their linkage to database records, also known as gene normalization (GN), is being developed as an alternative to the current time-consuming practice of manual extraction of names and has become an essential component of many text mining systems and database pipelines. For example, PubTator [3], incorporates GN to assist curation of genes in PubMed abstracts. Our group also uses GN of kinase and substrate mentions to integrate phosphorylation information from the text mining tool RLIMS-P [4] into iPTMnet (proteininformationresource.org/iPTMnet), a bioinformatics resource for protein post-translational modifications. GN also enables semantically refined literature searches [5] (e.g. retrieval of literature for a given protein in a particular taxon group), Thus, efficient and reliable GN systems can play a key role in the effort to link big data to knowledge.

GN involves two essential tasks: (i) the detection of gene mentions (GM) in text, and (ii) the association of database identifiers (IDs) to the detected genes. Both have significant challenges. Challenges for the GM task include: (i) textual variations of a given gene name in different articles (e.g., AtPLAIIA vs. AtPLA IIA), and (ii) polysemy, mentions that can refer to gene names or non-gene concepts or even common English words. (e.g., SNI can name both a gene and a non-gene entity) Challenges for the second database ID mapping task include: (i) identification of the species name, which is required for unique mapping to a species-specific database record and which may or may not be named in the same part of the text as the gene, and (ii) ambiguity, when multiple genes share the same name (e.g., SEN1 is used to name two Arabidopsis genes with UniProt ACs Q9M1E8 (tRNA-splicing endonuclease subunit Sen2-1) and A8MRI9 (Senescence-associated protein DIN1).

Gene normalization has been a key theme in several BioCreative Challenge Evaluations: BioCreative I [6] focused on the GN task for yeast, fly, and mouse genes, while BioCreative II [7] focused on the GN task for human genes, as illustrated below with a few examples. ProMiner [8] is a dictionary-based GN system involving manual clean-up of a dictionary and the inclusion of different biomedical dictionaries. GNAT [9] is a GN system encompassing four steps: named entity recognition for genes and species, validation of gene mentions, correlating gene mentions with species, and finally gene mention disambiguation. GeNo [10] tackles the GN problem by employing a carefully crafted suite of symbolic and statistical methods, and by fully relying on publicly available software and data resources. TF-IDF [11] weighting is used to calculate semantic similarity scores for resolving ambiguous names.

In BioCreative III [12], the GN task was further extended to cover genes of all relevant species in the literature corpora. Among the systems, Bhattacharya et al. [13] tried to associate a species name with a gene name by considering their proximity, which was achieved by choosing various window sizes for character boundaries. Dai et al. [14] employed a multi-stage GN procedure and selected dictionary entries from only the top 22 most common species in NCBI (from 7283 species) to speed up the GN process. A document-level gene normalization system, called GeneTUKit [15], employed features from the local context and the global context within the whole full-text article, and normalized genes of different species simultaneously. GenNorm [16] follows three steps: gene name recognition, species assignment, and species-specific gene normalization, and uses SR4GN [17] for assigning species to gene mentions. GenNorm has been widely used in text mining systems that require GN, such as in PubTator and in an event extraction pipeline [18]. The lack of limitation on species in GenNorm makes it suitable for such integration.

Despite this large body of work, gene normalization continues to be inadequate for certain taxonomic groups, particularly plants, where (i) there is a lack of common standard nomenclature across species [19–24], (ii) locus or ORF species-specific names are frequently used, and (iii) there is a high frequency of ambiguity in gene names because of the high number of paralogs in multigene families.

In this paper, we describe a plant-specific gene normalization system, called pGenN (pivot-based Gene Normalization). Because a dictionary is an essential component in GN and its quality significantly affects system performance, we built a large plant gene dictionary, providing extensive coverage while containing minimal false matches. We introduced an orthographic concept called pivot which captures the shared part of gene names of same family, and developed techniques centered around this idea that help in both the gene mention and the normalization processes. We also developed a method that can automatically generate an annotated gene mention corpus from scientific literature and employed this method to build a large gene mention corpus that includes text from the plant literature.

For evaluation purposes, we had to develop our own curated literature corpus given the limited availability of annotated plant literature in existing corpora. Evaluation on this corpus, which includes 104 Medline abstracts, shows that our system has better performance than GenNorm, a state-of-the-art system evaluated in BioCreative III, and the only publicly available GN tool for plant-related abstracts.

As GN tools can play an important role in text-mining pipelines, we demonstrated the integration of pGenN with RLIMS-P, a tool for curation of protein phosphorylation information. We used pGenN to normalize kinase and substrate mentions detected by RLIMS-P, illustrating that pGenN can successfully interoperate with other text mining modules for knowledge base curation.

Finally, we applied pGenN to all plant-related Medline abstracts to test its scalability. The results, which are updated monthly in sync with PubMed, are stored in a local database called pGenN_DB. The pre-processed results are publicly available via a web interface for multiple modes of querying and downloading of results.

## System and Methods

**Fig 1:**
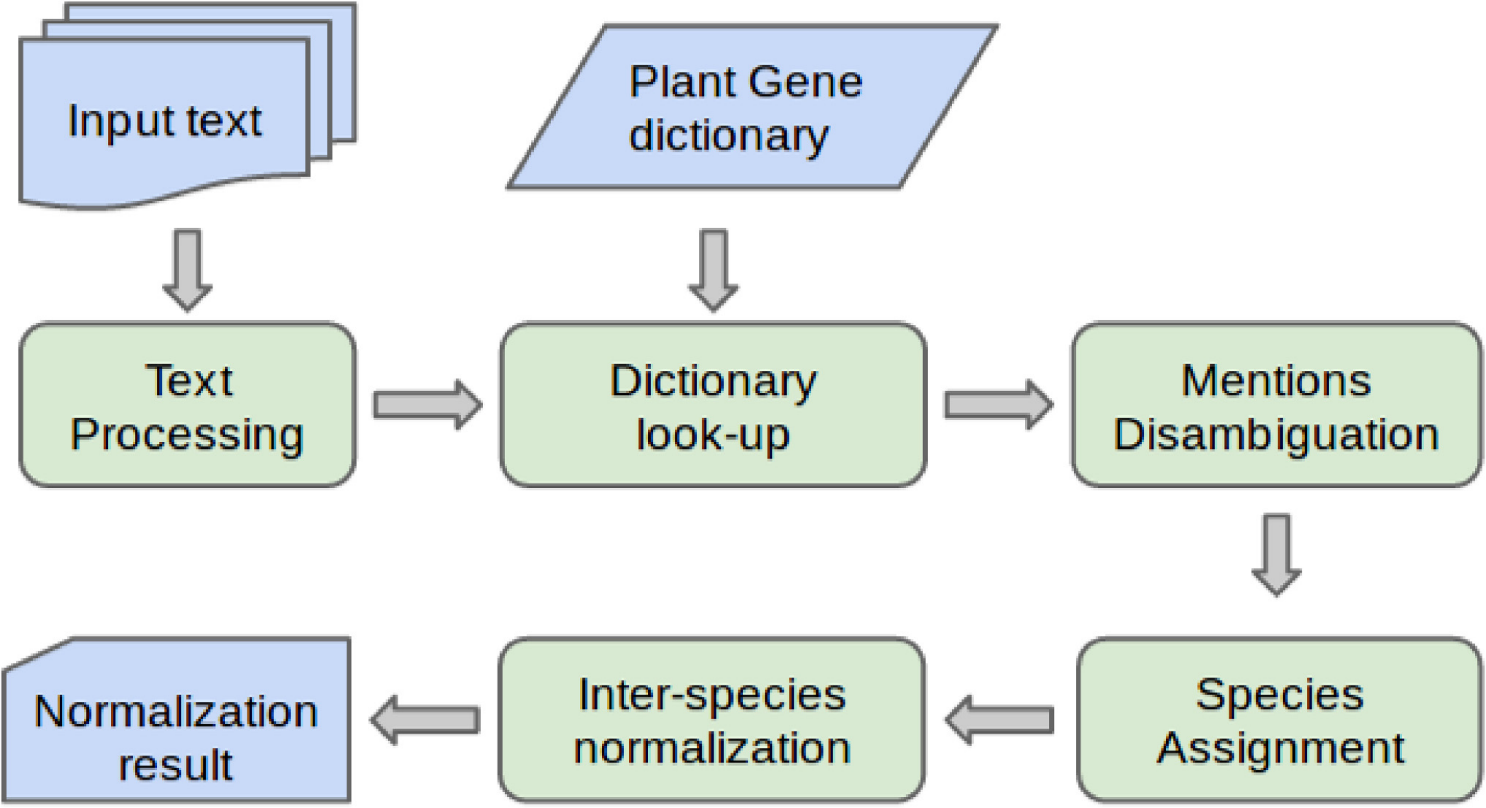
pGenN system architecture.

The overall architecture of pGenN is shown in Fig 1. The input text in Medline abstract format is broken up into sentences and then into individual tokens. Potential gene name candidates are first identified using a dictionary lookup. Next, a context-based disambiguation component decides which of these candidates correspond to actual gene mentions. Then, a species is associated with each detected gene mention. Using different features gathered from text and dictionary, pGenN completes the normalization process by assigning an identifier for a species-specific gene in the dictionary among those that matched the name found in the text.

### Text preprocessing

The system accepts as input a single or multiple PMIDs. Although it can process any text as input, some of the rules for species assignment were developed specifically for abstracts.

Given a list of PMIDs, the titles, abstract text, and the MeSH terms are extracted for each PMID. We use an in-house developed tool to split the abstract text into sentences and then tokenize the sentences. This tokenization is based entirely on orthographic features such as the combination of lower case followed by uppercase letters or presence of numerals and symbols. For example, terms such as AtAur1 and Mn(2+) are tokenized as “At Aur 1” and “Mn (2 +)” respectively. We then use an in-house developed tool to tag noun phrases in each sentence.

### Gene Dictionary: Creation and Use

Many existing gene mention recognizers (e.g., AIIAGMT [25] and BANNER [26]) do not use a dictionary-based approach. For example, BANNER uses conditional random fields (CRF) with orthographic, morphological and shallow syntax features. In contrast, we use a dictionary-based approach for the gene mention task. Dictionary matching is an essential part in the gene normalization task since the task of gene normalization is to link gene mentions with identifiers included in the dictionary. So even if a GN system uses a system such as BANNER to detect gene mentions, it would still need to match the detected mentions with a gene dictionary for normalization. Additionally, using a dictionary for gene mention detection avoids the problem of false negatives produced by the gene mention systems. Similar points have been observed earlier by others, e.g., Verspoor et al. [27].

**Fig 2:**
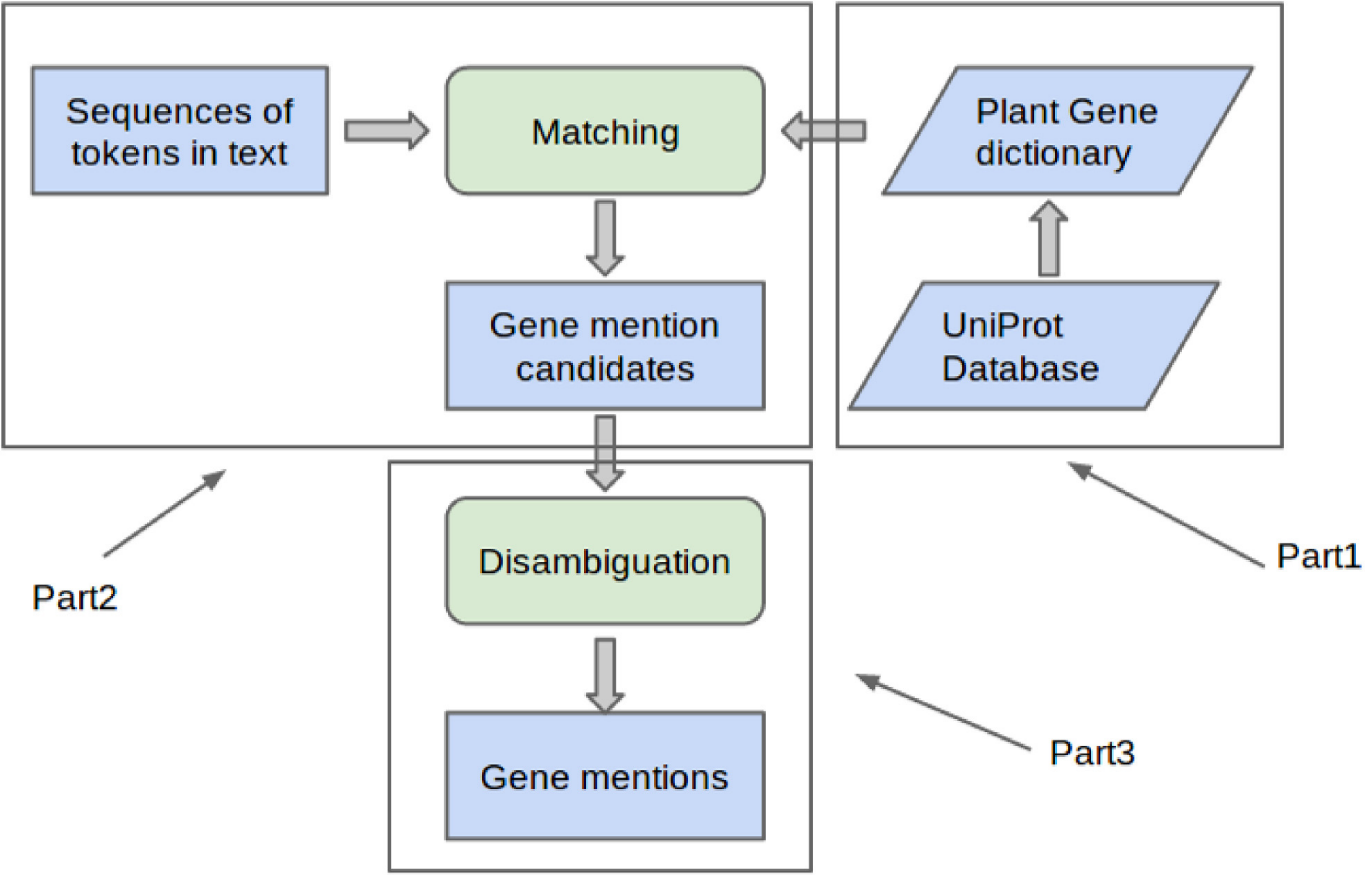
Components of dictionary-based gene mention detection.

There are three basic parts to a dictionary-based approach to gene mention detection, as depicted in Fig 2. First is the creation of an extensive dictionary. The dictionary must be as inclusive as possible so that gene mentions are not missed, but should also exclude names that can lead to incorrect matches. Second is the use of the dictionary in matching input text to identify gene names. Since gene names (especially short names) can also correspond to names of other biological entities such as diseases, matching against text only provides gene name candidates. A third step disambiguation is needed to determine whether a candidate corresponds to a gene mention or not. Typically, local context in the form of words close to the candidate is used for such disambiguation.

These three basic parts of the dictionary-based gene mention detection are discussed next.

### Creating a plant gene dictionary

The plant gene dictionary is based on the UniProt database. Of interest to us, UniProt protein entries contain protein accessions (ACs), protein names and gene names. Our dictionary is created by downloading all entries for all plant proteins in UniProt (http://www.uniprot.org/). Similarly to others who have built gene mention detection or normalization tools, we do not distinguish between genes and their protein products. Hence, we collected both gene and protein names from UniProt for all the plant gene/proteins. Each entry in the dictionary contains additional information, such as its species and synonyms (alternate names), along with the name.

While gene names from an entry are taken directly, protein names are further processed to extract parts that are likely to be found in text. Consider the following protein names “Pre-mRNA-splicing factor SLU7” (UniProt AC A2YQU8) and “Salt stress root protein RS1” (UniProt ID A2WMG6). In addition to the full names, we also include the shorter versions “SLU7” and “RS1” in the dictionary as they are often found in the abstract text without the preceding “descriptive” part. Both the shorter name version and the descriptive parts are useful, the former for the gene mention task whereas the latter can play an important role in the normalization task. In order to split the full name into these two parts, we look for protein names in a specific pattern: “word1 word2 … wordN f-term c-term”. The notions of c-terms and f-terms were introduced in [28] and further developed in [29]. An f-term comes from a small list of words such as gene, protein, factor and enzyme, which indicate that the entity is a gene or its product. Table 1 shows the regular expression we used for identifying gene f-terms. A c-term, on the other hand, is characterized by the presence of orthographic features such as capital letters, numerals and special prefix symbols, which indicates that the term is not a typical English word but likely to be a name.

**Table 1:** Regular expression for identifying gene f-terms.

/(gene|protein|factor|kinase|[^abehiou]ase|oncogene?|binder|globulin|tubulin|inter-?feron|lectin|galectin|globin|tinin|matin|ietin|tropin|zyme|kine|leukin|nogen|receptor|enzyme|hormone|protease|permease|nuclease|oncogene)$/

UniProt contains two types of entries: reviewed and unreviewed. The reviewed entries are curated by domain experts with information extracted from literature and curator-evaluated computational analysis and are assumed to be of high quality. Proteins and gene names in the reviewed entries include recommended name and synonyms from the literature and nomenclature standards, plus locus names and open reading frame names (ORFs) for gene names. The unreviewed entries, on the other hand, contain protein sequences (e.g., from translation of sequences in GenBank [30]) associated with computationally generated annotation and large-scale functional characterization. These entries have not been curated by human annotators. Protein and gene names in unreviewed entries come from direct submissions, propagation of annotation rules developed by UniProt, or other external sources. UniProt coverage for plant proteins in the reviewed section is limited; as an example there are only 446 entries for tomato proteins in the reviewed part, but 37,386 entries from this species in the unreviewed part. Thus, we also considered including gene and protein names from unreviewed entries in UniProt. A quick analysis of some unreviewed entries suggested that while the gene names were acceptable, the protein names should be used with caution because they are often very general. For example, in the unreviewed entry UniProt AC B6SS10, the Submitted (protein) full name is “Receptor kinase”. Using such names would lead to too many non-specific matches. As discussed above, the fact that the name ends with an f-term indicates that it is likely to be a generic name description rather than a specific name. Thus, from the unreviewed entries, we extract all gene names and only those protein names that include c-terms.

### Organization of the Dictionary

To discuss the organization of the dictionary, we first consider how a name like AtAur1 is stored. Every name extracted from UniProt is tokenized into three parts that we call the prefix, the pivot, and the suffix. The prefix represents the species, in this case, ‘At’ for Arabidopsis thaliana. A number of plant species follow a similar convention to indicate the species (two-letter abbreviation with the first letter uppercase and the second letter lowercase), but it is not universal. Several alternatives are shown in Table 2. Additionally there are species-specific locus ID conventions as well. The prefix is set to null if the species abbreviation is not included as part of the name. Because the species can be determined based on the UniProt record linked to the name, the prefix information is redundant and is not stored in the dictionary. The suffix includes numbers or Greek alphabet letters (or single uppercase alphabet letters corresponding to common Greek alphabet letters) that are found at the end of the names. Again the suffix is set to null if it is not present. We call the remaining part between the prefix and the suffix the pivot. Thus, in this case, ‘Aur’ is the pivot.

**Table 2:** Plant species prefix conventions.

2 letter prefix	First letter is upper case and second is lower case. e.g., “At” for “Arabidopsis thaliana”, “Os” for “Oryza sativa”.
3 letter prefix	Only for Brassica species. First letter must be upper case “B”, which is short for “Brassica”. Second and third letters are lower case. e.g., “Bra” for “Brassica rapa”, “Bni” for “Brassica nigra”.
4 letter prefix	For Latin binomial. The symbol for a binomial consists of the first two letters of the genus, plus the first two letters of the specific epithet. e.g., “PASM” for “Pascopyrum smithii”.
5 letter prefix	All the letters must be upper case, and the first three letters must be “VIT”. e.g., “VITVI” for “Vitis vinifera”.

**Fig 3:**
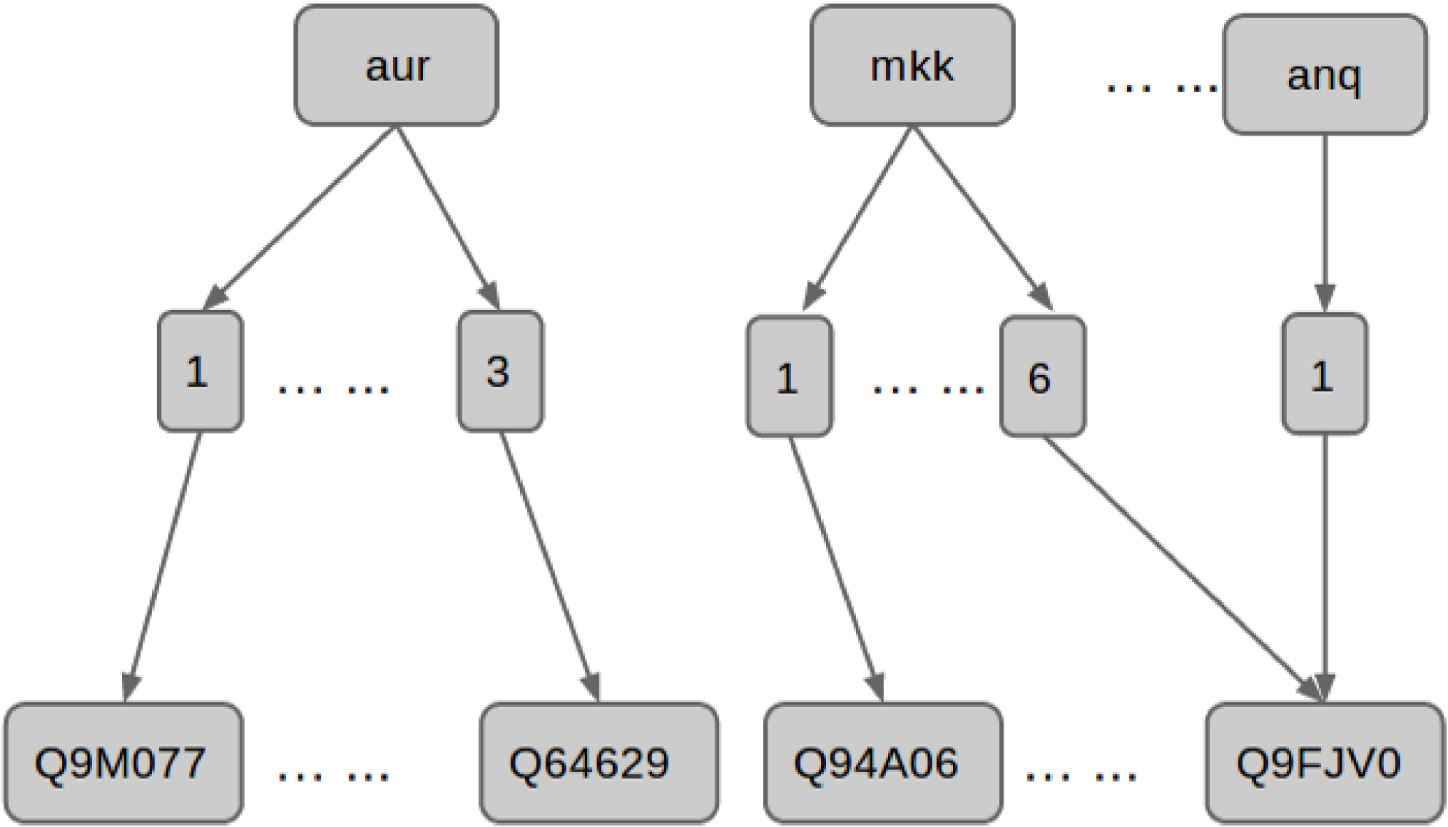
Pivot based plant gene dictionary structure.

The dictionary is organized hierarchically into three layers, as shown in Fig 3. At the top are nodes that are labeled with pivots. These pivot nodes have multiple child nodes where each child node corresponds to a different suffix. Each suffix node (together with its parent pivot node) corresponds to a specific gene name (recall the prefix nodes are not stored) and has as children UniProt AC nodes. In addition to storing the UniProt AC, the UniProt AC node has other information such as the species and all gene/protein names in the corresponding UniProt record. A UniProt AC node can have multiple parent nodes since many names may be listed in a given UniProt entry and hence share the same UniProt AC. Thus the dictionary is organized as a directed acyclic graph rather than a tree.

### Using the dictionary for Identifying Gene Mention Candidates

Consider 'AtAur1' appearing in text. It will be tokenized into prefix, pivot, and suffix ('At Aur 1') as stated earlier. This tokenization allows us to handle name variations similar to Joachim et al’s standardization [10] for dealing with name variations and will also handle cases mentioned by the regular expression approach of Jorg et al [9]. In our case, it allows matching the dictionary with text using a pivot based approach. Hyphens are treated in a special manner, during the dictionary matching process. All hyphens, except for the ones that appear between numbers (or single letters), are treated exactly like blank space during matching. On the other hand, hyphens between numbers (or single letters) are often used to indicate a range of values and are left as is during the matching. These are handled further by special rules (see Table 3).

**Table 3:** Rules for generating multiple candidates from a sequence of tokens.

**Parentheses rule.** e.g., from “interleukin (IL)-4”, get “interleukin-4” and “IL-4”.
**Suffix in conjunction rule.** e.g., from “Protein kinase C alpha, beta, and gamma”, get “Protein kinase C alpha”, “Protein kinase C beta”, and “Protein kinase C gamma”. This rule will also cover cases like “ERK 1/2”, where “ERK 1” and “ERK 2” will be generated.
**Suffix in range rule.** e.g., from “ERK 1–8”, get “ERK 1”, “ERK 2”,. . ., “ERK 8”.

The occurrence of 'Aur' in text will match a corresponding pivot node in the dictionary. The next token in text ‘1’ matches one of the dictionary entry suffixes under that pivot node. Hence the tokens “Aur 1” can now be considered a candidate gene mention, and several UniProt ID nodes for different species are linked to the suffix node with this candidate gene mention. Note that preceding token “At” in text is not matched since that information is not included in the dictionary. However, the presence of this two letter token in text is noted as a string matching a species prefix and will be used for normalization (species assignment).

Although Table 2 provides the conventions for naming plant gene names, sometimes authors do not follow them exactly and occasionally, gene names mentioned in text may not fit the patterns mentioned in Table 2. As examples, ‘AtAur1’ can appear as ‘atAur-1’ or ‘Ataur-1’. These mentions will not be matched if we fail to detect the prefix. Thus, if a possible gene name (e.g., a c-term) does not match with the dictionary, its initial letters will be extracted to verify if it is a plant species prefix. If this is the case then the rest of the name will be used to match with the dictionary.

Some minor extensions are added to this simple matching approach to enhance the performance. The first extension is a longest match strategy: if one match occurred inside another, only the longer match will be kept. For example, ‘Bcl-2’ in 'Bcl-2-associated athanogene 7' can be matched to a name in the dictionary, but it will not be kept as candidate gene mention because it is nested in another longer match ('Bcl-2-associated athanogene 7').

Another extension involves creation of multiple candidates from a sequence of tokens. Consider, for example, the input sequence 'Interleukin (IL)-4'. Triggered by the parenthesis, the system will form two sequences 'Interleukin– 4' and 'IL- 4' and both of them will be matched against the dictionary using the pivot based approach. The input sequence is considered a match even if only one of the sequences produced from it matched with the dictionary. Due to the parenthesis in the sequence, our heuristic rule will assume both sequences represent the same gene candidate and hence both will be normalized to just one UniProt entry. Other rules allow for matching a single sequence of tokens with multiple entries. Consider the input sequence: 'Protein kinase C alpha, beta, and gamma'. After matching the pivot 'Protein kinase', during matching of the suffix, we note the conjunction and hence produce three sequences (‘Protein kinase C alpha’, ‘Protein kinase C beta’, ‘Protein kinase C gamma’). However, in this case, unlike in the parenthesis case, it is not assumed that the multiple sequences represent the same gene candidate. Two similar rules are used to handle input sequences like ERK 1–8 and ERK 1/2. The rules for generating multiple candidates from a sequence of tokens are summarized in Table 3.

Finally, we noted that pivot-based matching allowed for detection of gene mentions beyond the names found in the dictionary. For example, consider the occurrence of 'FLORAL BINDING PROTEIN 11' in PMID 12481066. While the dictionary derived from UniProt does not have this name, there are other names such as 'FLORAL BINDING PROTEIN 1' sharing the same pivot. As the only difference between these two names is in the suffix, and the suffixes are of the same type (numbers), pGenN proposes 'FLORAL BINDING PROTEIN 11' as candidate gene mention. Of course 'FLORAL BINDING PROTEIN 11' cannot be normalized and hence this detection is only useful if the tool is being used independently as gene mention detector. We also note that pivot-based matching allows for detection of family names. For example, consider the occurrence of 'ERF' in PMID 25641039, which matches with a dictionary pivot node (‘ERF’), but has no suffix. All the names in the dictionary linked with this pivot node have suffixes, such as ‘ERF1', ‘ERF2’ and ‘ERF14’, so we will predict that 'ERF' is a gene family mention.

### Using Context to Determine Gene Mentions

As discussed earlier, dictionary look-up alone is not sufficient to detect gene mentions. For example, consider the following sentence from PMID 6857705: “injection of the purified toxins are 91 (SN1) and 71 (SN2) micrograms/kg mouse”. The name candidate “SN1” matches a pivot and suffix in the dictionary, but it is not a gene mention.

In general, a candidate can have a gene sense or a non-gene sense. In this section we discuss how we decide if each candidate is a gene mention or has a non-gene sense. First, we apply some heuristic rules to decide whether a candidate gene mention is an actual gene mention or not. Next, we apply a support vector machine (SVM) model for predicting gene or non-gene sense. It is customary for sense disambiguation to be handled by considering context in the form of words that appear nearby, and hence the words appearing nearby are used as the features for SVM learning. One of the distinguishing aspects of our sense disambiguation approach is that we use only immediate local context (within two words to the left or right) as features. We believe that such an approach has potential to achieve high precision but can also lead to low recall. To alleviate this problem we have developed a technique to propagate gene context to other instances of candidate mentions. This technique is assisted by the idea of pivots we have introduced here. Additional motivation for and details of these steps are described next. The automated method for creating a large gene mention corpus which includes plant related literature is discussed in a later section.

### Rule-based disambiguation

The first rule is applicable for a candidate mention that matches a dictionary full name. Since long names are assumed to not correspond to a non-gene concept, these full name mentions are directly considered as gene mentions, and therefore are not subjected to the processes described below.

Second, candidate mentions can sometimes be inferred to be family names or complex names, and hence excluded on that basis. Rules for filtering out these types of mentions are shown in Table 4.

**Table 4:** Rules for filtering out family and complex name.

If NAME appears at the end of a noun phrase, and the noun phrase starts with “a” or “another”, then NAME will be considered as family name and filtered out.
If NAME is followed by words “family” or by an f-Term in plural form, then NAME will be considered as family name and filtered out.
If NAME appears at in the end of a noun phrase, and the NAME is preceded by “subunits of”, then NAME will be considered as complex name and filtered out.
If NAME is followed by word “complex”, then NAME will be considered as complex name and filtered out.

Third, some language structures are used by the author to give additional descriptive information, hence they provide clear clues to disambiguate names as gene or non-gene. For example, in the sentence…flowering by repressing the transcription of FT, a flowering-integrator gene that encodes…’, the appositive of name ‘FT’, ‘a flowering-integrator gene …’, provides a strong clue that ‘FT’ is indeed a gene. The pGenN method detects two language structures, acronym and appositive. Acronyms are detected using an in-house build acronym detector, which is based on Stanford acronym detection algorithm [31], and appositives are detected using the text mining system iSimp [32]. Rules that are based on these two language structures, as well as two other rules (Dictionary matched name in relation and Synonym) used for disambiguation purposes are shown in Table 5.

**Table 5:** Rules for disambiguation of name as gene or non-gene.

Acronym rule: If an acronym pair is detected, and the full name matches with the gene dictionary or ends with an F-term, then the short name will be assigned a gene sense.
Appositive rule: If NAME has an appositive, and the appositive ends with an F-term, then NAME will be assigned a gene sense.
Dictionary matched name in relation rule: If two or more names are matched with the dictionary and they appear together in a conjunction with other candidate mentions, then all the names will be assigned a gene sense.
Synonym rule: If NAME1 and NAME2 are synonyms (matched with the same dictionary entry), and they appear in the same article, then both NAME1 and NAME2 will be assigned a gene sense.

### SVM-based disambiguation

We use the presence of nearby words to determine if the candidate mention is an actual gene mention or represents some other type of entity. We restrict the use of context to words appearing immediately to the left or right of a candidate. Such use of immediate context is motivated by an attempt to achieve high precision, since words adjacent to the candidate are likely to be related to candidate, whereas words that are further away may pertain to some other entities and hence not be necessarily related to the candidate.

Support vector machines (SVMs) [33] are supervised learning models and are commonly used for classification. We employed SVM-light [34], an implementation of the SVMs, using default parameter settings and a linear kernel to learn the disambiguation model.

The six features used for learning correspond to: a single word appearing to the immediate left or immediate right (LI-SW and RI-SW), a single word appearing within two words to the left or right (LW2-SW and RW2-SW), and two words appearing to the immediate left or immediate right (LI-TW and RI-TW). For example, given the sentence “Mutants lacking jasmonate synthesis or response had decreased MYB21 expression and …”, the feature attributes associated with the candidate “MYB21” are ‘LI-SW: decreased’, ‘LI-TW: had decreased’, ‘LW2-SW: decreased’, ‘LW2-SW: had’, ‘RI-SW: expression’, ‘RI-TW: expression and’, ‘RW2-SW: expression’, and ‘RW2-SW: and’. Using an automated method discussed below, we developed a large gene mention corpus which covers plant related literature and annotated the named entities as gene or non-gene. We collected all words and bigrams that appeared next to the annotated named entities to form a feature set. To enhance effective learning, we removed the common English words in the feature set using a stop word list, and selected only the features with a frequency greater than 30. Finally, we got a feature set with 1739 features.

### Propagating Contexts

**Fig 4:**
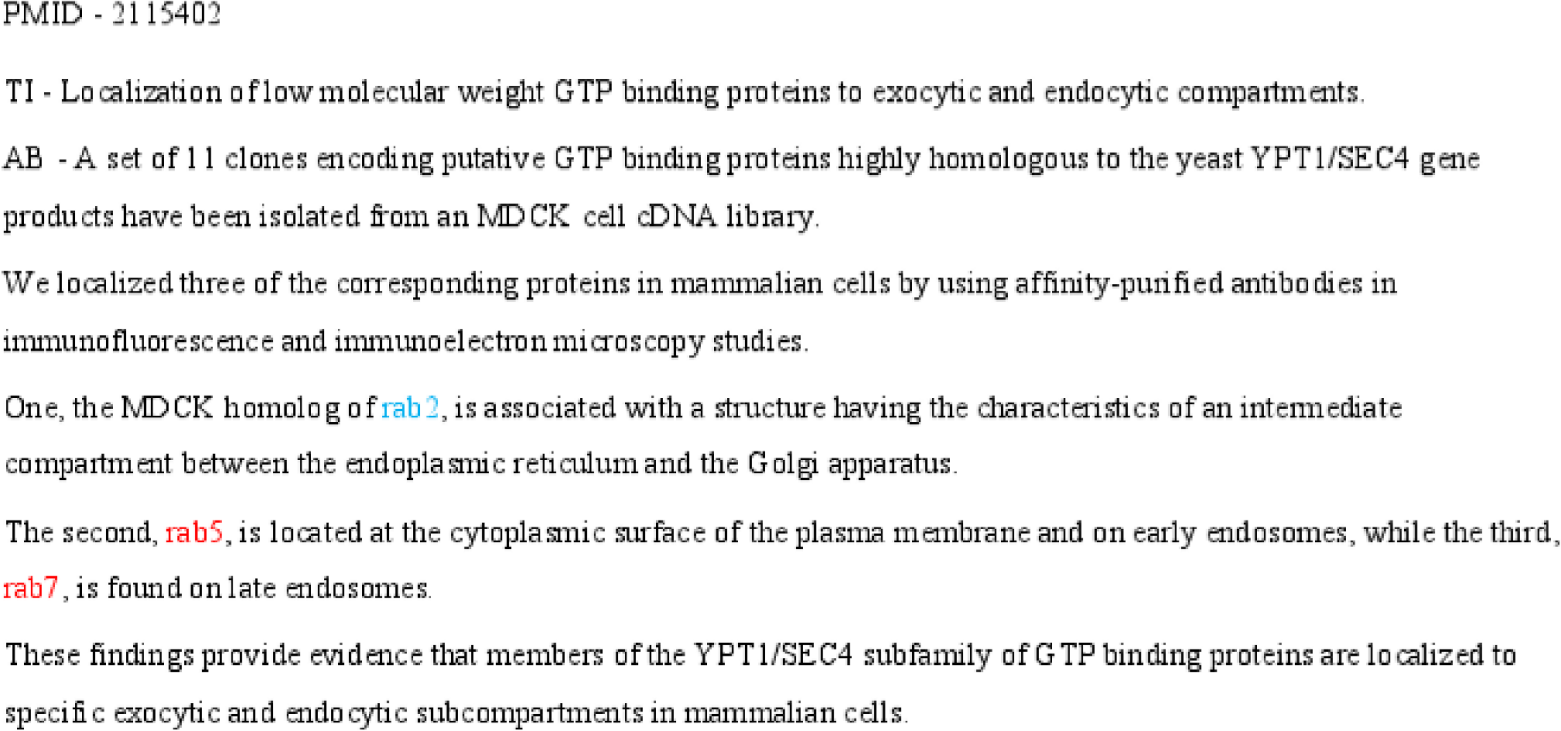
Example for ‘uni-pivot gene sense assumption’.

Despite developing a large training corpus for training the SVM-based disambiguation model, it still may suffer from low recall. Many occurrences of gene names might not have words immediately to the left or right for us to assign a gene sense with confidence. Consider the occurrence of “rab5” (or “rab7”) in Fig 4. Clearly words nearby are not sufficient to clearly assign a gene sense (or a non-gene sense). However, if there is another occurrence of “rab5” (or “rab7) in the same abstract, which had a clear-cut evidence for the disambiguating model to assign a gene sense, then we assume that both the occurrences will have the gene sense. This assumption, that multiples occurrences of the same candidate name within the same abstract have the same sense, has a long history in natural language processing and can be called as the single sense per name per document rule. But, in this abstract (Fig 4) there is no other occurrence of “rab5” (or “rab7”) to apply this rule. However, the notion of pivot allows us to generalize the rule to single sense per pivot per document rule. By this rule, the occurrence of rab2 (which shares pivot with rab5 and rab7), which can be clearly disambiguated as having a gene sense by the SVM model on the basis of its context to the left, will allow occurrences of rab5 and rab7 also to have a gene sense. Note that the single sense per pivot per document rule subsumes the single sense per name per document rule for propagating context.

Finally, we found that the single sense per pivot per document could improve gene mention detection even when the gene names were not present in the dictionary. Of course, since the additional names are not in the dictionary, they cannot be normalized and so only the gene mention detection part is improved.

In spite of our efforts to increase the application of context based disambiguation by such propagation, there could still be instances of candidates which do not have context features to assist the disambiguation. Based on our experience of analyzing a significant number of Medline abstracts, those candidates should be assigned a non-gene sense.

### Species Detection and Assignment

A gene name matched in text can be associated in the dictionary with several UniProt records corresponding to different species. So the next task we considered was the assignment of a species to a matched instance based on the text around it. This involved using a recognizer of species mentions in text and connecting each gene mention with the species mentions that were detected in the document.

SR4GN is a well-known recognizer of species names in text that assigns species to gene mentions and has been adopted by other gene normalization tools such as GenNorm. SR4GN uses a species name dictionary for recognizing species and uses a few rules to assign species to gene mentions. However, we found we could not use it for our purpose. First, while it uses prefixes found in gene/protein names to assign species, the prefixes are limited to those of a handful of species that did not include the range of plant species of interest to us. Second, SR4GN employs additional heuristics such as the presence of words in the document like “cohort” or “ferment” to assign species when none are detected in the document. Again these heuristics do not appear to extend to plant species. Finally, SR4GN always tries to assign some specific species to every gene mention, whereas our analysis of plant literature suggested that there were several cases where the gene mentioned did not correspond to a specific species but rather had a more generic usage (i.e., species independent). Thus, we concluded that we needed to develop a method to detect and assign species to gene mentions that extends the rules developed for SR4GN.

The detection of species is handled by two components. The first component performs a dictionary matching, using a species dictionary built from NCBI Taxonomy. Because authors often abbreviate the species name by using the initial letter for the genus (e.g. A for Arabidopsis) followed by a period and then the species name (thaliana). Accordingly, the dictionary was extended by adding these type of names. e.g., new name ‘A. thaliana’ was generated from ‘Arabidopsis thaliana’. The second component identifies prefixes in gene and protein names that conform to plant species prefix conventions (same conventions as shown in Table 2), as these could indicate the presence of a species. e.g., ‘At’ for ‘Arabidopsis thaliana’, ‘Zm’ for ‘Zea mays’, ‘Os’ for ‘Oryza sativa’.

The gene mentions are associated with the species based on the following rules. Note that the rules are ordered based on our confidence in their precision. This ordering is used to determine which rule should be used in case more than one of them applies. If a species is assigned for a particular mention based on some rule numbered x, then no other rule lower in the ordering (i.e., numbered greater than x) can over-ride that assignment.

1. Prefix. If a gene mention has a species prefix, we assign the species based on the prefix. e.g., ‘AtAurora1’ would be assigned the species ‘Arabidopsis thaliana’. This rule is similar to Rule R1a in SR4GN.
2. Same noun phrase. If a gene mention and species are in the same noun phrase, we assign that species to the gene mention. e.g., ‘Arabidopsis TOC1/PRR1 gene’ would be assigned the species ‘Arabidopsis thaliana’. There is no direct analog in SR4GN, but this rule is inspired by Rule R1b.
  This rule also considers the case where the species appears in a prepositional phrase that is attached to the noun phrase containing the name (as in ‘SEX4 from Arabidopsis’). This is an improvement over SR4GN Rule R1b in cases where multiple species and multiple genes are mentioned in the same sentence. For example, in PMID 23879260, the sentence 'The predicted protein for CpPG1 has 416 amino acids, with a high homology to other pollen PGs, such as P22 from Oenothera organensis (76%) and PGA3 from Arabidopsis thaliana (73%)'. Using Rule R1b, SR4GN would assign PGA3 with species Oenothera organensis whereas our rule will correctly associate PGA3 with Arabidopsis thaliana.
3. Species-free. If a gene mention is in the ith sentence, and the first species mentioned in the article is in jth sentence and i< j, then we assume that the gene mention doesn’t belong to any particular species. SR4GN does not have a rule corresponding to this; SR4GN will always assign the gene mention with a species as long as any species is found in the article.
4. Single species. If only one species is mentioned in the abstract, then all the gene mentions in this abstract will be connected with this species. This is similar to SR4GN Rule R1c.
5. Species consistency. If a gene mention with name ‘NAME1’ has been assigned a species in one of the previous sentences, then this occurrence of ‘NAME1’ will be assigned the same species used for the closest occurrence. This rule captures the intuition that authors typically do not switch species without some explicit notification. There is no corresponding rule in SR4GN.
6. Species in the same sentence. A gene mention is assigned to a species that occurs in the same sentence. If there are multiple species in the same sentence, pick the one to the left. This is the same as SR4GN Rule R1b.
7. Species in the previous sentence. A gene mention is assigned to a species that occurs in the previous sentence. If there are multiple species in the previous sentence, then this rule is not applied. There is no corresponding rule in SR4GN.
8. Major species. Species in the title and in the MeSH terms are considered to be the major species of the article. A gene mention is assigned to the major species. If there is more than one major species, then this rule is not applied. This is similar to the idea of focus species in SR4GN, except that we have introduced several other rules, which have no corresponding rules in SR4GN, prior to the application of this rule.

### Intra-species normalization

Once we have detected gene name mentions and we have assigned a species to each gene mention, we then use the dictionary to obtain a list of candidate identifiers (UniProt ACs) for that name and species pair. To complete the gene normalization task, we need to choose one of the candidate identifiers. Like other gene normalization tools, we use the context of the gene mention and information about the identifiers obtained from gene/protein resources to make the choice. Some gene normalization system, e.g., GNAT, use additional information, such as the GO terms associated with the identifier for disambiguation. Like GenNorm, pGenN only considers the names associated with the identifiers from the dictionary. However, unlike GenNorm, pGenN uses text close to the gene mention for disambiguation, whereas GenNorm only use the names detected in text for disambiguation.

As for previous steps, we have a number of rules that are applied hierarchically, where the order of the rules is based on our confidence in them.

The first type of context we considered is in the form of the acronym, appositive, or relative clause attached to the gene mention, or words appearing in the same noun phrase containing the gene mention. Again as noted before, this type of context is typically used to introduce descriptions relevant to the entity. Hence we believe words in this type of context are the strongest clues for disambiguation. Words in this type of context are compared to the words associated with each candidate identifier in the dictionary, and the identifier with the most word matches is chosen as the normalization result. However, the context discussed above might not always exist for all gene mentions. If we cannot disambiguate (i.e., identify a single identifier) based on this type of context, we will consider words in the same sentence as context.

So far, we have described the method as though we normalize each gene mention independently of all others. This is not strictly true. If a name appears multiple times in a document and the same species is assigned to these gene mentions, we wish to assign the same identifier to all of them. Also, since we used only immediate context to match with information of candidate IDs, many mentions might not occur with disambiguating words in such context. To ensure that all the occurrences of the same name were assigned with the same ID, and to address the potential low recall, we treated all occurrences of the same gene as a single instance where we combined the context for all the occurrences of the same name.

We generalized this idea of sharing context information even if different occurrences of the same name had different species assigned to them. However, in this case, although the context was shared, different identifiers would be assigned, because the genes belong to different species.

Finally, we generalized this context sharing strategy to names with the same pivot: all the names with the same pivot would share the same context, because we believe the same abbreviation in one article will have the same expansion. We have never observed any case where one abbreviation had multiple expansions in one article. However, to make our rule more robust, if we detected different expansions for the same abbreviation, then this context sharing strategy would not be used.

### Developing annotated corpus for GM task

Recall that we needed an annotated gene mention corpus for machine learning to determine if the candidate mention is an actual gene mention or represents some other type of entity. The current existing corpora either tend to be organism specific, domain specific, or not large enough to cover a wide range of immediate gene contexts. e.g., the GENIA corpus [35] is focused on a subset of human hematology, the PENNBIOIE [36] corpus is on oncology, the BioCreative 1 GM corpus [37] contains only 15,000 sentences, the BioCreative 2 GM corpus [38] contains only 20,000 sentences (15,000 of which were used previously in BioCreative 1). A machine learning-based disambiguation model trained on those corpora using only immediate contexts as features is quite likely to suffer from low recall. A similar observation has been made by Wermter et al. [11]. Also, since our task is focused on plant species, a gene mention training corpus that includes text from the plant literature will be the best choice for our system.

To develop a new large gene mention corpus that covers substantial plant literature would be difficult if it relies solely on expert annotation. Hence we developed an automatic method to create annotated gene mention corpora. Since our method only requires raw text as input, we can efficiently create very large annotated data sets. In addition, our method can be applied to other settings beyond our particular use here. However, we need to take care to ensure that there is little noise in the data.

We retrieved all of the Medline abstracts containing one or more gene short names that appear on a list of ~50,000 gene short names from our plant dictionary. Next, we used an algorithm modified from the Stanford acronym detector to detect the full name-short name pairs in these abstracts and picked the ones which have short names appeared in the dictionary. Note that the appearance of these names in the abstracts does not mean that they refer to genes necessarily. Our task was to identify which amongst those pairs represented genes and which did not. For this purpose, we considered the extracted full name and assigned it a gene sense only if the full name appeared in the dictionary with the short name or it ended with an f-term. On the other hand, if the full name does not end with an f-term nor has any word in common with corresponding full names in the dictionary, then a non-gene sense was assigned. Otherwise, it was left unannotated. As an example, the pair, “ataxia telangiectasia mutated (ATM)”, was left unannotated. The full name does not meet the requirement for gene sense assignment. However since there is a partial match with a full name (which includes ataxia and telangiectasia) corresponding to “ATM” in the dictionary, it was not assigned non-gene sense either.

These simple rules allowed us to identify instances of names from a gene dictionary that had gene or non-gene sense with high degree of confidence. We manually analyzed a sample of 40 full name-short name pairs where the short name appears in in our plant dictionary and found the rule marked 36 as having gene sense and remaining four were left unannotated. All 36 names marked with gene sense were indeed gene mentions. It turns out that even the four unannotated instances were gene mentions.

Once the mentions were assigned a gene or non-gene sense, all occurrences of the same name within the abstract were also tagged. Finally, all of the positive full names and short names were replaced with the string “NAMEP”, and all the negative full names and short names were replaced with “NAMEN”. By applying this method, we obtained a large annotated corpus and used the first 200,000 abstracts with 157,336 gene positive instances and 120,308 gene negative instances as our gene mention corpus.

Although this method might not identify all the gene mentions in text, our key hypothesis is that we can still automatically obtain a sufficiently large training corpus that covers a wide variety of contexts by applying this rule to a very large number of abstracts. Also, since all name mentions are not annotated with gene or non-gene sense, it cannot be used for evaluation of gene mention detection. Additionally, this corpus can only be used for learning the context of gene and non-gene mentions, but not words appearing within the names since the mentions are replaced by strings “NAMEP” and “NAMEN”.

### Evaluation Method

Since we are not aware of any existing corpus annotated for plant gene normalization, we developed our evaluation corpus in-house. The abstracts used in this set were identified by searching Pubmed using the query: ("Proteins"[MeSH] OR "Genes"[MeSH]) AND "Viridiplantae"[MeSH]. One hundred and four abstracts were selected from the retrieved abstracts, with a selection process that attempted to ensure coverage of a range of different gene names, different species, and different publication years. The annotation was completed by one of the co-authors who is a senior bio-curator. This annotation was done independently of the system development and then the system output was compared against the manual annotation for obtaining the evaluation results. Altogether 195 instances of UniProt accession numbers-PMID pairs were annotated from the 104 abstracts. The evaluation set is publicly available at proteininformationresource.org/iprolink.

As noted earlier, most of the existing gene normalization tools were designed for non-plant species and hence are not appropriate for comparison. Some of these tools are also not publicly accessible. Thus we were able to compare with GenNorm only. Recall GenNorm is the gene normalization component used within PubTator, which is applied on all of Medline, and hence not restricted by species. GenNorm is available through PubTator and obtained the best results in the BioCreative III gene normalization task. It uses SR4GN to assign species to gene mentions in text. Again, the species are not limited, and include plant species. We believe that the comparison must be viewed with two points in consideration. As noted before, some of the rules of SR4GN are specific for human and a few other non-plant species. Second the quality of normalization results will depend on the gene name dictionary used. GenNorm uses EntrezGene to create its dictionary and our cursory observations lead us to believe that UniProt has more comprehensive coverage of plant species than EntrezGene.

The system performance was computed using the standard measures of precision, recall and F-measure. Gene/protein mentions linked to accession numbers of non-plant genes were not considered. pGenN may return multiple UniProt ACs for one gene mention, due to: (1). the UniProt entries are almost identical except for the subspecies designation. e.g., Oryza sativa subsp. indica (Rice), or Oryza sativa subsp. japonica (Rice). (2). the UniProt entries are redundant, corresponding to reviewed and unreviewed entries for the same entity. Errors that originated from either of these two reasons were ignored. GenNorm normalizes genes to EntrezGene identifiers. To compare the performance with GenNorm, we used the ID mapping tool provided by UniProt to convert these identifiers to UniProt ACs.

### Medline abstracts processing and interactive web Interface

To verify the scalability of pGenN and to develop a large body of pGenN results that we could make accessible to the community, we processed all plant-related abstracts that we identified in Medline using the broad query, ‘plant[MeSH] AND hasabstract[Text]’. We used ‘hasabstract[Text]’ to filter out the PMIDs which only have titles. The 444,211 abstracts which were returned by PubMed for this query were processed by pGenN and the results were stored in a local database which we call pGenN_DB. We intend to update pGenN monthly in sync with PubMed.

**Fig 5:**
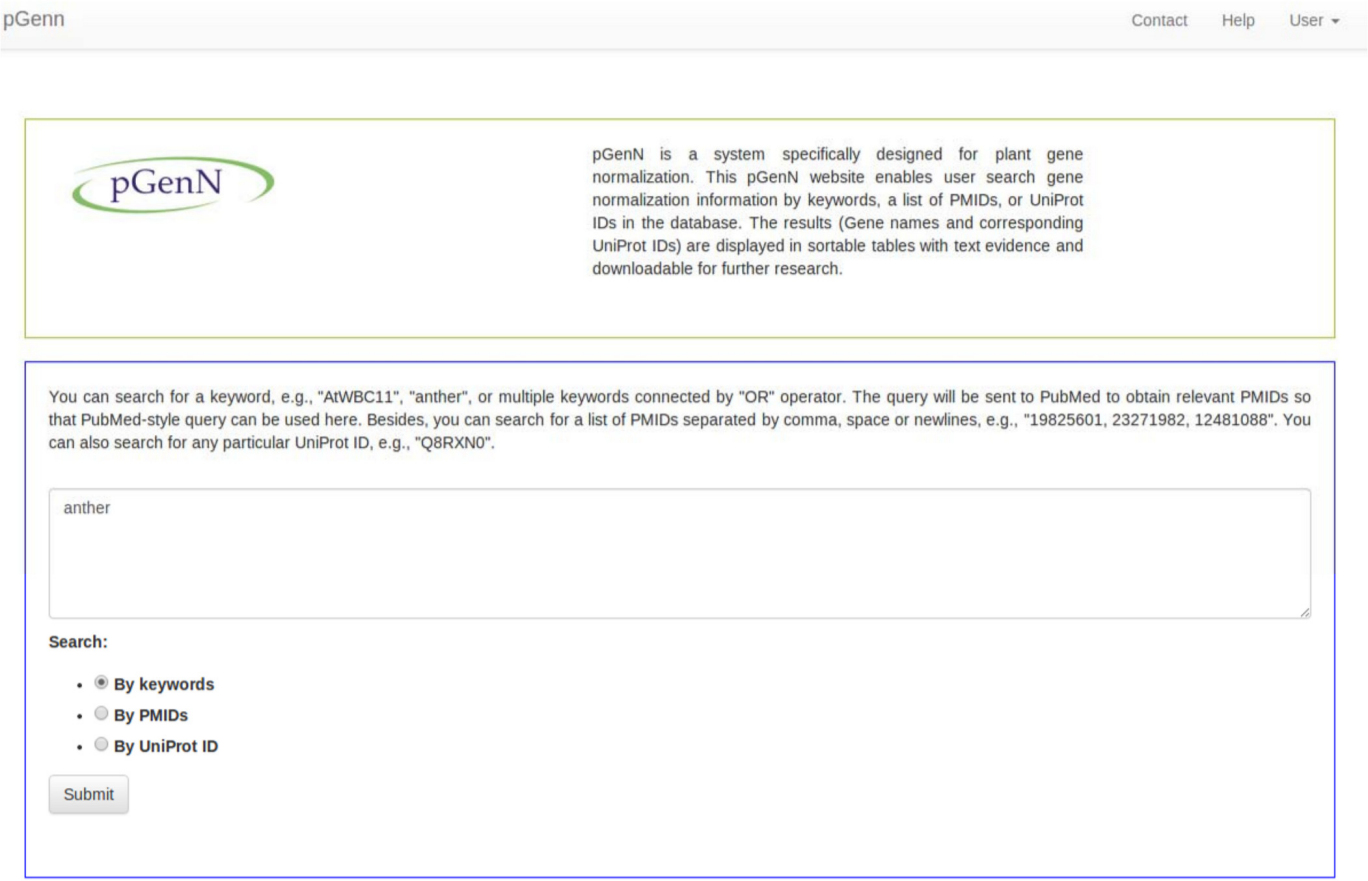
Screenshot of pGenN Interface.

A web interface (proteininformationresource.org/pgenn) has been developed to enable community access to the plant gene normalization results in pGenN_DB. Fig 5 shows the homepage of the pGenN interface where users can search for plant gene normalization results. Users can query using a list of PMIDs, UniProt ACs, or any keyword(s) in a PubMed-style query (boolean operators 'AND', 'OR', 'NOT' are allowed). For example, a researcher interested in the topic of plant anthers can use a query with “anther”. The query is used to obtain a list of PMIDs from PubMed and the results from the subset of abstracts in pGenN_DB are then retrieved. Because the results in pGenN_DB are preprocessed, the response is fast.

**Fig 6:**
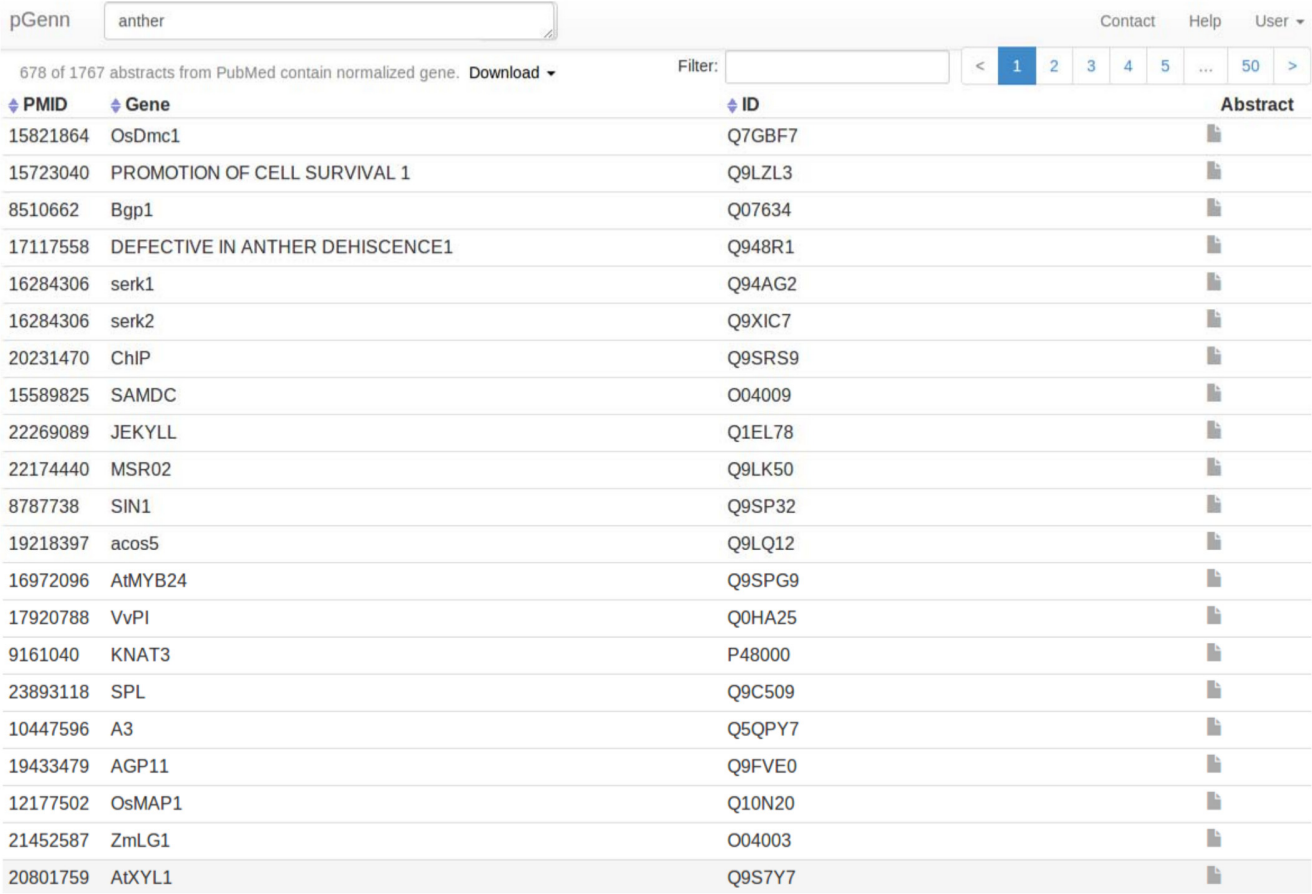
Screenshot of pGenN result table.

Fig 6 shows the first page (of over 50 pages) of results obtained using the query “anther”. Results are displayed in a table with columns for PMID, gene name, UniProt AC and Entrez gene ID. One gene name with multiple mentions within a single PMID will only be shown once in the table. Because users may be more interested in the most recent papers, or in some particular genes, we added a sort function, that will sort results by PMID, by Gene name, by UniProt AC, or by Entrez gene ID.

**Fig 7:**
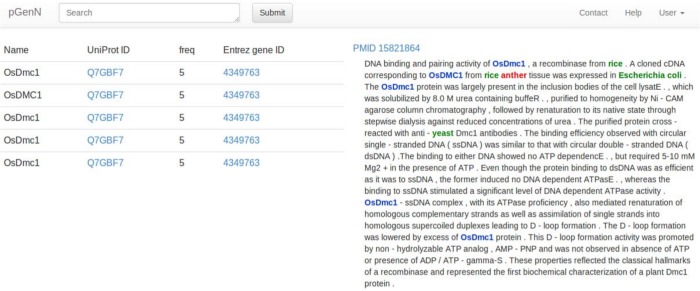
Screenshot of pGenN text evidence page.

Users can view the evidence tagged abstracts for each gene normalization result by clicking the Abstract button in any table row. On the left half of the text evidence page (Fig 7), every gene mention, as well as its corresponding UniProt AC and Entrez gene ID are shown. The UniProt ACs and Entrez gene IDs are hyperlinked to their original entries in UniProt and EntrezGene. As also shown in Fig 7, in the right side of the text evidence page, the Medline abstract is shown with all gene mentions highlighted in blue, species highlighted in green, and query keywords (if searched by keyword) highlighted in red.

### Use Case Study: Gene Normalization of Large-Scale Text-Mining Results

Text mining tools that extract gene/protein-based information from text can be run on a large scale and the information gathered can be stored formats amenable to searching or incorporation into curation pipelines. However, it is even more useful if the gene/proteins can be normalized to unique database (e.g., UniProt) identifiers. As a use case, we applied pGenN to plant-related phosphorylation information obtained through large-scale text-mining using RLIMS-P. RLIMS-P, which extracts information about kinase, substrate, and site from text, has been run on the entire set of Medline abstracts. For determining the effectiveness of plant gene normalization in conjunction with this tool, we selected a set of 87 abstracts using the query “brassinosteroid signaling” in which RLIMS-P extracted at least one kinase or substrate. Brassinosteroids are a class of plant hormones that regulate gene expression via a signaling cascade that involves multiple phosphorylation events [39]. The quality of gene normalization by pGenN was compared with a manual annotation of kinase and substrate occurrences in these abstracts.

## Results and Discussion

### Evaluation results on in-house corpus

Our evaluation set contains 104 abstracts that contain 195 instances of UniProt accession number-PMID pairs. Currently, pGenN does not process information in full-text articles, so our evaluation was limited to abstracts only. Table 6 shows the precision, recall and F-measure for both pGenN and GenNorm.

**Table 6:** Performance of pGenN & GenNorm on in-house plant corpus.

	Precision	Recall	F-value
pGenN	90.9%	87.2%	88.9%
GenNorm	57.6%	39.0%	46.5%

Table 6 shows that pGenN achieves high precision and recall. We analyzed the false positive and false negatives of pGenN to learn how different components of pGenN contributed to the errors.

The within-species normalization component worked surprisingly well, considering we use limited features (based only on names in the dictionary) for disambiguation. This component contributed to error in only one instance, resulting in both a FP and a FN. Many of the errors were due to incorrect assignment of species. This situation is consistent with an observation in Wei et al. [16] that accuracy of species assignment is critical for overall performance on the gene normalization task. When the species is incorrectly assigned, clearly a wrong accession number will be assigned. This not only results in false positives but false negatives as well. An example of an incorrect assignment of species comes from PMID 11197326: “OsMADS14 and -15 are highly homologous to the maize MADS box gene ZAP1 which is an orthologue of the floral homeotic gene APETALA1 (AP1).” Based on the same sentence rule, the closest species mention “maize” is assigned to “AP1” incorrectly. Other species assignment errors were also similar and involved mentions of homologs. The second source of errors is due to the dictionary based gene mention detection. For example, in PMID 16455357, the mention “Ljcen1” did not match with the correct dictionary entry because we failed to detect the species prefix “Lj”, which is short for the species “Lotus japonicus”. This was due to the fact that the gene name cen1 did not start with an upper-case letter as expected based on other plant species naming conventions. Some of the errors were due to failure to capture all variations of a gene name in the dictionary. In PMID 17114582, the text includes a mention of "MtHAP2-1". However, the name in UniProt is "HAP2.1" (UniProt AC A4ZVU9), and we had not accounted for this variation.

### Statistics of Large-Scale Processing

Table 7 shows the statistics of the processing of 444,211 plant-related Medline abstracts that were obtained by the PubMed query “plants[MeSH] AND hasabstract[Text]”. In these abstracts, 313,334 gene mentions from 58,301 abstracts were normalized. These corresponded to more than 27,000 unique UniProt accessions and a little over 112,000 pairs of abstracts and UniProt accessions.

**Table 7:** Statistics of pGenN large-scale processing of plant Medline abstracts.

# of abstracts processed	444,211
# of abstracts which are pGenN positive	58,301
# of gene mentions normalized	313,334
# of unique UniProt ACs obtained	27,496
# of PMID–UniProt AC pairs obtained	112,053

Since the 444,211 abstracts resulted from a search for plant-related articles in general, we wanted to see if we would obtain a larger proportion of abstracts that are pGenN positive (i.e., contain at least one normalized gene mention) if we modified the query to a more gene-specific query ‘plants[MeSH] AND (gene[MeSH] OR protein[MeSH]) NOT animal[MeSH] AND hasabstract[Text]’. ‘NOT animal[MeSH]’ was included to rule out articles about pharmaceutical use of plant products for animal disease. This query resulted in 97,611 abstracts. Table 8 shows that in this subset, nearly 37% of the abstracts were pGenN positive. To get a better sense of pGenN’s ability to identify relevant abstracts, we manually analyzed 100 PMIDs from the pGenN negative papers (the remaining 65%). We found that only 7 of these 100 abstracts contained gene mentions that can be normalized to UniProt ACs (false negatives). In five cases, pGenN properly detected the gene mention but not the species and in the remaining two cases, the gene mention was not detected. Our analysis of the remaining 93 abstracts showed that most of the pGenN negative abstracts did not have any gene name mentioned in the abstract although some had indications that the full-length articles might contain some gene mentions (e.g., PMID 24097262 talks about a family of transcription factors and PMID 21653281 discusses gene expression patterns during seed coat development in Arabidopsis). There are three abstracts that included plant gene mentions that could not be resolved to any UniProt plant AC since UniProt did not contain entries for those genes (although it included entries in other species for the same name).

**Table 8:** Statistics of pGenN processing of gene/protein-related subset of plant Medline abstracts.

# of abstracts processed	97,611
# of abstracts which are pGenN positive	36,261
# of gene mentions normalized	224,273
# of unique UniProt ACs obtained	20,986
# of PMID–UniProt AC pairs obtained	74,069

### Use Case Study: Normalization of Genes/Proteins Related to Phosphorylation in the Brassinosteroid Signaling Pathway

The case study involved a query “brassinosteroid signaling” and the use of those abstracts for which RLIMS-P, an existing phosphorylation information extraction tool, extracted at least one kinase or substrate. These 87 abstracts were manually annotated for gene normalization, yielding 153 instances of UniProt accession number-PMID pairs, where the accession numbers corresponded to a kinase or a substrate. Table 9 shows pGenN’s performance against this gold standard as well as comparison with GenNorm.

**Table 9:** Performance of pGenN & GenNorm on use case data set.

	Precision	Recall	F-value
pGenN	97.9%	93.5%	95.6%
GenNorm	93.5%	66.0%	77.4%

These results are better for both systems than those obtained for the evaluation data set. Perhaps the biased nature of the abstracts and the limited number of proteins may explain the better performance on this set.

The results suggest that it would be fruitful to incorporate pGenN for normalizing RLIMS-P extraction results. We aim to now use pGenN in normalizing kinase and substrate mentions in all plant-related abstracts from which RLIMS-P has extracted a mention of phosphorylation events with a kinase and/or phosphorylated substrate. The results of the entire plant-based text-mined phosphorylation results will be accessible via iPTMNet (http://proteininformationresource.org/iPTMnet/).

## Conclusion

We have presented here a gene normalization system, pGenN, which is designed to normalize plant genes. The development of pGenN involved design of new methods in all phases: dictionary-based gene mention detection, species assignment and normalization. In developing pGenN, we have introduced a new concept of pivot that has been used in all phases of the normalization and helps improve the recall. We have also developed a new method for generating a large training set for the gene mention task that requires minimal manual intervention. We believe this method can be used generally to learn context for gene mention detection in specific domains. We conducted an evaluation of pGenN on a plant-centric set of abstracts and the results show that pGenN can be used to meet the need for a plant gene normalization system, achieving an F-value of 88.9% on our in-house annotated plant gene normalization corpus. Based on the case study we conducted, we believe that pGenN can be integrated into text mining pipelines and we intend to use pGenN as part of iPTMNet, a resource for protein post-translational modification that draws, in part, on information gathered by text mining tools. Additionally, pGenN has been used to process a comprehensive set of over 440,000 plant related Medline abstracts. The pre-processed results have been stored in our local database, pGenN_DB, and. can be searched, sorted and downloaded via a web interface, found at http://proteininformationresource.org/iPTMnet/.

In the future, we plan to expand our system to cover non-plant species. While many methods that have been introduced here should be directly applicable to other species, we need to investigate how well our algorithm for species assignment to gene mentions, which was developed based on analysis of plant-related literature, generalizes to non-plant species. Our aim is to use pGenN in many text mining applications and steps are already underway to integrate it into iPTMNet. We also plan to enhance our automatic training corpus creation approach for detecting other biomedical named entities. Finally, we would like to investigate the usage of pivot based dictionary matching to enhance two aspects of curation of the Protein Ontology (PRO) [40]: (1) Detecting gene family names to enhance coverage of protein family classes and (2) Normalizing family protein names to PRO IDs when terms already exist in the ontology.

## Supporting Information

S1 File Plant gene normalization evaluation corpus (ZIP)
